# Cyclooxygenase-2 mediated synergistic effect of ursolic acid in combination with paclitaxel against human gastric carcinoma

**DOI:** 10.18632/oncotarget.21576

**Published:** 2017-10-06

**Authors:** Xian Xu, Guo-Qin Zhu, Kai Zhang, Yi-Chan Zhou, Xiao-Lin Li, Wei Xu, Hao Zhang, Yun Shao, Zhen-Yu Zhang, Wei-Hao Sun

**Affiliations:** ^1^ Department of Geriatric Gastroenterology, The First Affiliated Hospital to Nanjing Medical University, Nanjing 210029, China; ^2^ Department of Gastroenterology, Nanjing First Hospital, Nanjing Medical University, Nanjing 210006, China; ^3^ Department of Geriatric, Huadong Hospital Affiliated to Fudan University, Shanghai 200040, China

**Keywords:** ursolic acid, paclitaxel, gastric cancer, apoptosis, cyclooxygenase-2

## Abstract

Ursolic acid (UA) induces apoptosis in gastric cancer cells by inhibiting cyclooxygenase-2 (COX-2). Paclitaxel (PTX) is an important chemotherapy agent used to treat solid tumors. We evaluated the *in vitro* antitumor activity of UA in combination with PTX against gastric cancer cells and investigated the mechanisms underlying the combined effects. A cytotoxicity test and flow cytometry were utilized to study the effects of UA and PTX on proliferation and apoptosis, respectively. To further elucidate the mechanism, Western blot analysis was used to assess changes in the expression of a series of related proteins, including COX-2, proliferating cell nuclear antigen (PCNA), Bcl-2, and Bax. UA and PTX dose- and time-dependently inhibited BGC-823 and SGC-7901 gastric cancer cell proliferation. Combined delivery of UA and PTX synergistically reduced cell proliferation and induced apoptosis in these cells by lowering COX-2, PCNA, and Bcl-2 expression and by increasing Bax expression. These results indicate that the synergistic inhibition of proliferation and induction of apoptosis by UA and PTX may be induced by reducing COX-2 expression in gastric cancer cells.

## INTRODUCTION

Gastric cancer, one of the most common cancers worldwide, is the second leading cause of cancer deaths [1, 2]. An estimated 679,100 cases of gastric cancer were diagnosed and 498,000 deaths occurred in China in 2015 [3]. Early detection and radical surgery have increased the survival of patients with gastric cancer. However, the prognosis of patients in advanced stages of gastric cancer is unfavorable because of the lack of efficient adjuvant therapy [4, 5]. Novel molecules from medicinal plants that have higher efficacy and lower toxicity when combined with traditional chemotherapeutics can be identified [6, 7].

**Figure 1:**
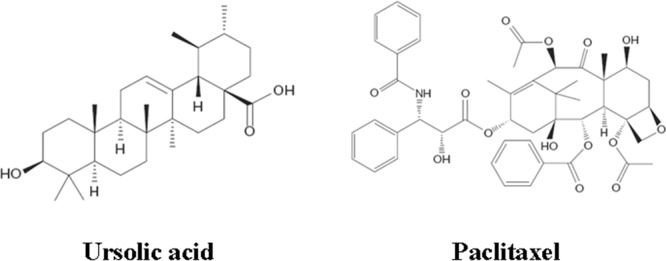
The chemical structures of UA (molecular formula C_30_H_48_O_3_, and 456 711 g/mol molecular weight) and PTX (molecular formula C_47_H_51_NO_14_, and 853.918 g/mol molecular weight).

Ursolic acid (UA) (Figure 1), a pentacyclic triterpenoid, has been identified from a variety of fruits, such as apples and cranberries, and from the herb basil. UA inhibits the growth of gastric cancer cells and colon cancer cells [8–10]. In animal models, UA inhibited tumorigenesis [11] and suppressed tumor invasion and metastasis [12, 13]. We found that UA induces apoptosis via down-regulation of cyclooxygenase-2 (COX-2) in gastric cancer cells [10]. COX-2 is expressed in gastric cancer and is a factor in cancer cell proliferation and apoptosis, cancer invasiveness, and metastasis [14, 15]. Studies have shown that COX-2 inhibition by selective COX-2 inhibitors or small interfering RNA (siRNA) suppresses cell growth and leads to apoptosis in human gastric adenocarcinoma cells [16, 17]. Moreover, studies have demonstrated the chemosensitization effect of UA combined with commonly used chemotherapeutics such as gemcitabine, cisplatin, and capecitabine [18–20]. For example, Prasad et al. [18] reported that UA inhibits the growth of human pancreatic cancer and enhances the antitumor potential of gemcitabine.

Paclitaxel (PTX) (Figure 1) is a promising cytotoxic agent. It acts through tubulin binding, thus disrupting DNA duplication [21]. PTX has been approved by FDA as a first-line chemotherapy regimen for a series of solid tumors, including gastric cancer [22, 23]. However, like most chemotherapeutic regimens, PTX has serious side effects, including peripheral nervous system toxicity [24], because of insufficient selectivity.

A phase II trial reported that the addition of a selective COX-2 inhibitor might enhance the response to preoperative PTX and carboplatin in patients with non-small-cell lung cancer [25]. Ferrandina et al. [26] reported that the expression of COX-2 is closely related to the sensitivity to PTX. Therefore, combined application of COX-2 specific inhibitor is a promising strategy to achieve better efficacy of PTX [27].

In the present study, human gastric cancer cell lines BGC-823 and SGC-7901, in which COX-2 is expressed [14], were utilized to investigate the antitumor effects of UA in combination with PTX in human gastric cancer *in vitro*. In addition, COX-2 expression, proliferating cell nuclear antigen (PCNA), and apoptosis-related proteins were detected, further elucidating the possible mechanism underlying the antitumor effects of UA and PTX in gastric cancer.

## RESULTS

### Effects of UA and PTX on BGC-823 and SGC-7901 cell proliferation

**Figure 2:**
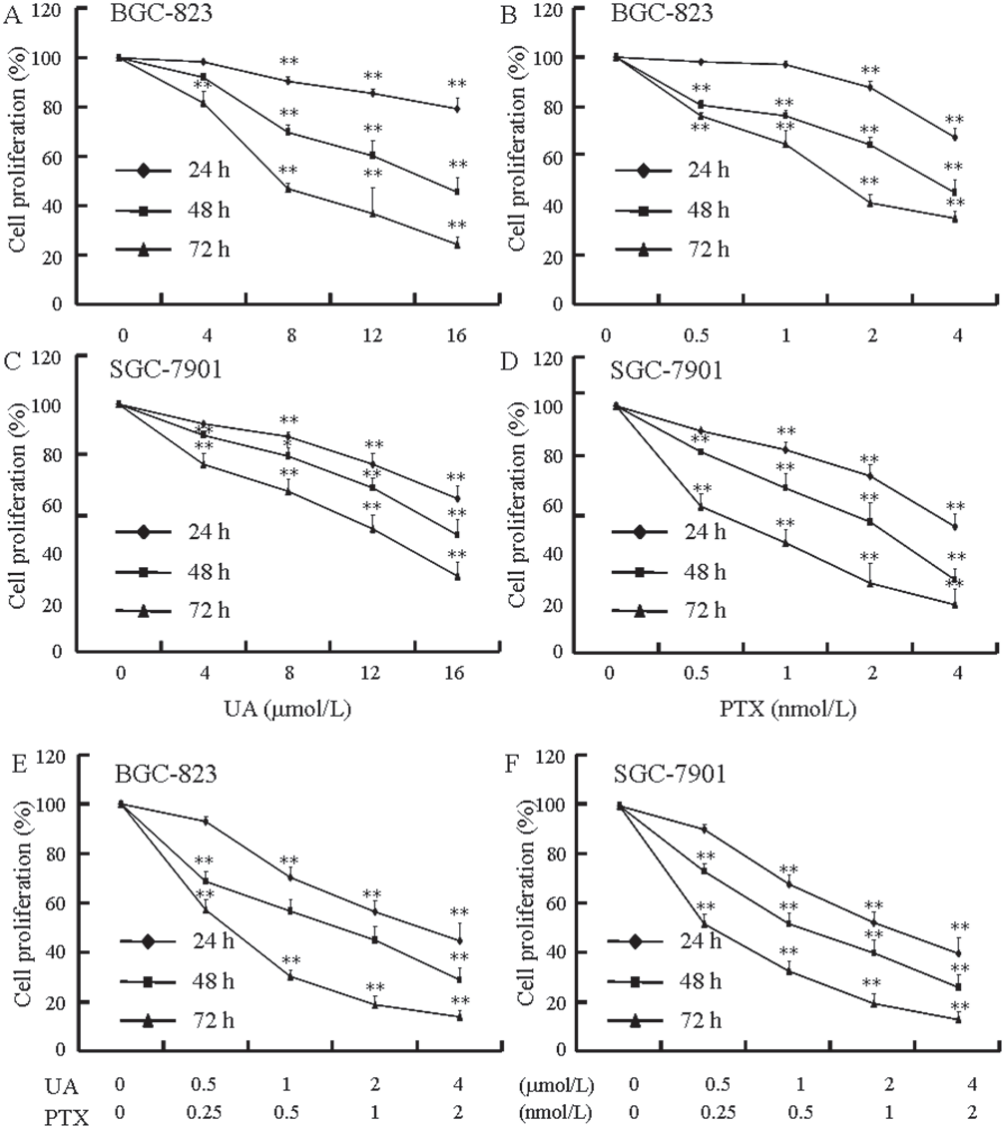
Effects of UA and PTX on BGC-823 and SGC-7901 cell proliferation **(A)** Dose-dependent and time-dependent effects of UA on BGC-823 cell proliferation. **(B)** Dose-dependent and time-dependent effects of PTX on BGC-823 cell proliferation. **(C)** Dose-dependent and time-dependent effects of UA on SGC-7901 cell proliferation. **(D)** Dose-dependent and time-dependent effects of PTX on SGC-7901 cell proliferation. **(E)** Effects of UA in combination with PTX on BGC-823 cell proliferation. **(F)** Effects of UA in combination with PTX on SGC-7901 cell proliferation. BGC-823 and SGC-7901 cells were treated with various concentrations of UA and PTX, or the combinations of the two agents for 24, 48, or 72 hours. The results are expressed as the percentages of the control. Data are shown as mean ± SD from three independent experiments. ^**^P< 0.01 vs. control.

**Figure 3:**
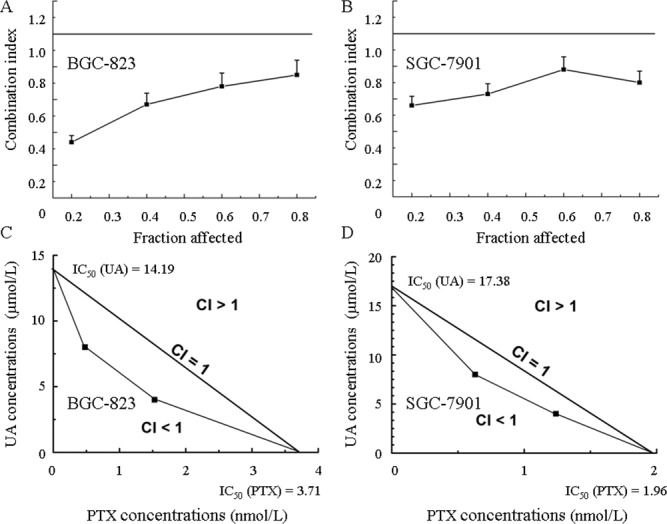
The combination of UA and PTX is synergistic in inhibiting proliferation of BGC-823 and SGC-7901 cells **(A** and **B)** The combination index for each fraction-affected value (Fa) was calculated by use of the Chou-Talalay method. **(C** and **D)** Representative isobologram of the treatment of BGC-823 and SGC-7901 **cells** with a combination of UA and PTX.

The MTT assay is commonly utilized to evaluate the survival of cells exposed to different therapeutics. UA (Figure 2A and 2C) and PTX (Figure 2B and 2D) both time-dependently and dose-dependently inhibit cell proliferation. Figure 2E and 2F show that simultaneous administration of UA and PTX increases cytotoxicity more than either of the single agents alone. As shown in Figure 3A and 3B, the combination-index (CI) method was utilized to evaluate the synergy of UA and PTX *in* inhibiting *proliferation* of BGC-823 and SGC-7901 cells. The CI was less than 1 at each fraction, which indicates a synergistic anti*proliferation* effect of UA and PTX. In addition, isobologram analysis was performed to confirm the results in Figure 3A and 3B. When the dose of UA is fixed at 4 and 8 μmol/L for the combination, the IC_50_ value of PTX at two combinational doses, which was lower than PTX used alone, is located inside the triangle area of Figure 3C and 3D. Therefore, Figure 3 reveals a significant synergistic effect of UA and PTX in BGC-823 (Figure 3A and 3C) and SGC-7901 cells (Figure 3B and 3D).

### Effects of UA and PTX on the apoptosis of BGC-823 and SGC-7901 cells

**Figure 4:**
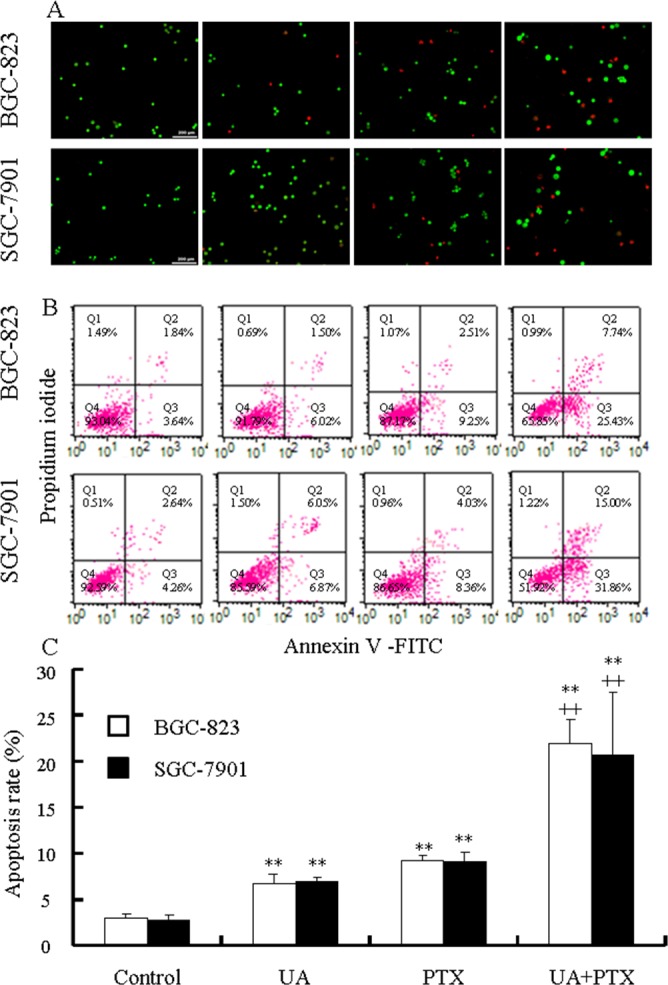
Effects of UA and PTX on the apoptosis of BGC-823 and SGC-7901 cells Cells were treated with 12 μM UA, 2.5 nM PTX, or both for 48 hours. **(A)** Morphological changes in apoptotic cells were examined by fluorescence microscopy after AO/EB dual staining. **(B)** Flow cytometry-based annexinV-FITC/PI labeling of apoptotic cells. **(C)** The histogram at the bottom represents the mean ± SD of apoptosis rates obtained from three independent experiments. ^**^P < 0.01 vs. control and ^++^*P* < 0.01 vs. UA or PTX.

BGC-823 and SGC-7901 cells were treated with UA (12 μmol/L), PTX (2.5 nmol/L), or both for 48 hours and stained with acridine orange/ethidium bromide (AO/EB) to identify different stages of apoptosis through apoptosis-associated characteristics of cellular membranes. Normally, early apoptosis is characterized by granular or crescent shaped yellow to green AO staining, and late apoptosis is characterized by concentrated and asymmetrically localized orange nuclear EB staining. Necrotic cells are indicated by uneven orange-red fluorescence. As shown in Figure 4A, the control cells exhibited homogenous chromatin morphology, with no sign of apoptosis. UA-treated or PTX-treated cells exhibited early and late apoptosis as well as necrosis. Combined treatment induces more apoptosis or necrosis than either of the two agents alone. In addition, apoptosis was evaluated by annexin V/*propidium iodide* (PI) double staining and flow cytometry. In Figure 4B, the lower right panels correspond to cells with apoptosis showing bright fluorescein isothiocyanate (FITC) and dark PI signals. Both UA and PTX led to apoptosis in BGC-823 and SGC-7901 cells, and the combined application of the two agents had stronger apoptotic induction (Figure 4C).

### Effects of UA and PTX on the expression of COX-2, PCNA, Bcl-2, and Bax in BGC-823 and SGC-7901 cells

**Figure 5:**
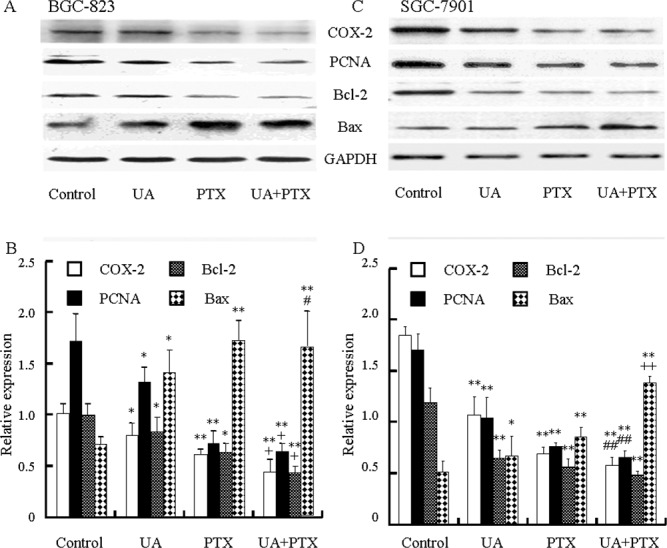
Effects of UA and PTX on the COX-2, PCNA, Bcl-2, and Bax expression in BGC-823 and SGC-7901 cells Cells were treated with 12 μM UA, 2.5 nM PTX, or both for 48 hours. **(A** and **C)** COX-2, PCNA, Bcl-2, and Bax expression was detected by Western blot analysis. **(B** and **D)** The histogram represents the relative expressions of COX-2, PCNA, Bcl-2, and Bax compared with GAPDH. Each data point represents the mean ± SD from three independent experiments. ^*^P < 0.05, ^**^P< 0.01 vs. control; ^+^*p* < 0.05, ^++^*p* < 0.01 vs. UA or PTX; and ^#^*P* < 0.05, ^##^*P* < 0.01 vs. UA.

To study the effect of UA and PTX on cell proliferation and apoptosis induction, expressions of COX-2 (involved in proliferation and apoptosis), PCNA (involved in proliferation), Bcl-2, and Bax (involved in apoptosis) in BGC-823 (Figure 5A and 5B) and SGC-7901 cells (Figure 5C and 5D) were evaluated by Western blot analysis. The results showed that UA or PTX suppressed COX-2, PCNA, and Bcl-2 expressions but increased Bax expression in BGC-823 and SGC-7901 cells. UA in combination with PTX inhibited COX-2, PCNA, and Bcl-2 expression and induced Bax expression more than either agent alone.

## DISCUSSION

Chemotherapy is the main treatment option for patients with advanced stage gastric cancer. Although PTX is frequently used in patients with gastric cancer [28, 29], this agent has limited efficacy and has side effects [24].

Combined application of different chemotherapeutics is currently the main treatment strategy in clinical settings. Studies have demonstrated the potential of herbal medicine as a novel adjuvant for chemotherapy that effectively enhances the antitumor effect of chemotherapeutics (PTX, cisplatin, etc.) [30, 31]. Our study has shown that UA induces apoptosis of gastric cancer cells by down-regulation of COX-2 [10]. Moreover, *in vivo* evaluation in a xenograft model of gastric cancer confirmed the antitumor effect of UA [32], which suggests that UA might have the potential of chemosensitization. The rationale for the combination of UA and PTX is based on the synergism between the two agents. Moreover, the synergism is dependent on the inhibition of COX-2 expression by UA, which is a factor in the reversal of PTX resistance, as reported previously [26].

Therefore, we investigated whether UA derived from various plant sources has potential either alone or in combination with PTX in the treatment of gastric cancer. He et al. [33] reported that UA showed synergistically hypoglycemic acitivity with other agents by scavenging excess intracellular reactive oxygen species. No synergistic antitumor effects of UA with other chemotherapeutics have been reported. Results from the current report have proved the dose-dependent and time-dependent inhibitory effects of UA on cell proliferation, which is in accordance with previous studies [32, 34]. UA in combination with PTX exerted synergistic anticancer effects *in vitro*. No study has previously reported the synergistic antitumor effect of UA combined with PTX. This study is the first to demonstrate the chemosensitization effect of UA in gastric cancer.

To elucidate the possible mechanism by which UA inhibits the proliferation of gastric cancer cells, expression of proliferating cell nuclear antigen (PCNA) was detected by Western blot analysis. Our results showed that UA and PTX reduced PCNA expression in BGC-823 and SGC-7901 cells. PCNA, one of the nuclear proteins, is produced in late G1 and S phases of the cell cycle and is often used as an indicator of changes in cellular proliferative status [14, 35]. PCNA expression is an indicator of early changes in cellular proliferation and possibly promotes the inhibitory effect on cell proliferation by UA, PTX, or both. In our previous study, co-treatment with NS-398, a selective COX-2 inhibitor, reversed the inhibitory effect on PCNA expression in human pancreatic cancer cells [35]. The suppressive effect on cell proliferation by UA or PTX is possibly induced by the prevention of COX-2 inducing PCNA expression in gastric adenocarcinoma cells.

Our previous study showed that UA can decrease the expression of COX-2 in gastric cancer cells [10]. Both *in vitro* and *in vivo* results from previous studies have shown that the elevated expression of COX-2 counteracts the apoptosis induced by the increase of Bcl-2 expression [36, 37]. The treatment with NS-398 is effective in inhibiting the expression of Bcl-2 while promoting the expression of Bax in human gastric cancer cells [17]. These results indicate that apoptosis induced by UA, PTX, or both is most likely linked to the down-regulation of COX-2 protein in gastric cancer cells. Studies have reported that inhibition of COX-2 reverses the resistance of several types of cancer cells to PTX [38, 39]. Additionally, studies have shown that inhibition of COX-2 expression leads to apoptosis in cancer cells by stimulating pro-apoptotic proteins [40, 41]. Thus, down-regulation of COX-2 and the consequent activation of apoptosis is a way to sensitize gastric cancer cells to PTX.

This study demonstrates that UA, a naturally occurring triterpenoid, can enhance the antitumor effect of PTX on gastric cancer cells through the down-regulation of COX-2, the decrease of the ratio of Bcl-2/Bax, and the consequent induction of apoptosis. Although UA has not been approved for clinical application, it is an effective and low-toxic adjuvant for PTX in the treatment of gastric cancer.

## MATERIALS AND METHODS

### Drugs and reagents

UA, PTX, Methyl thiazolyl tetrazolium (MTT), dimethyl sulfoxide (DMSO), and 4′,6-diamidino-2-phenylindole (DAPI) were purchased from Sigma-Aldrich Chemical Company (St. Louis, MO, USA). Stock solutions of UA and PTX were made in DMSO at 0.1% final concentration and sterilized by passage through a 0.22-μm filter (Immobilon, Millipore Corp., Bedford, MA, USA), diluted with culture media before use. RPMI-1640 medium, fetal bovine serum (FBS), and penicillin/streptomycin were purchased from Gibco BRL (Grand Island, NY, USA). Other reagents were of analytic grade and obtained from Nanjing Chemical Reagent Company (Nanjing, China), unless otherwise described.

### Cell lines and culture conditions

The poorly differentiated human gastric cancer cell line BGC-823 and the moderately differentiated gastric cancer cell line SGC-7901 were obtained from the Cell Bank of Type Culture Collection of the Chinese Academy of Sciences, Shanghai Institute of Cell Biology (Shanghai, China). The cells were cultured in RPMI-1640 medium supplemented with 10% FBS, 100 U/mL penicillin G, and 100 μg/mL streptomycin at 37°C in a humidified incubator with 5% CO_2_.

### Methyl thiazolyl tetrazolium cell viability assay

BGC-823 and SGC-7901 cells were seeded onto 96-well microplates at a density of 5 × 10^3^ cells per well and incubated overnight in 10% FBS medium. The cells were then treated with different concentrations of UA or PTX in serum-free conditions. After incubation for 24, 48, and 72 hours at 37°C, cell viability was determined by the MTT assay as described in our previous study [17, 35]. Each experiment was performed in triplicate. The results were presented as the percentages relative to the control cells treated with 0.1% DMSO. The 50% inhibitory concentration (IC_50_) was calculated by use of the dose-response curve.

### Analysis of combined effects

The combined effects of UA plus PTX were assessed by the combination-index (CI) method [42] and isobologram analysis [43]. On the basis of the dose-response curves utilizing the MTT assay, the CI is defined by the following equation: CI = (D)_1_/(Dx)_1_ + (D)_2_/(Dx)_2_ + (D)_1_(D)_2_/(Dx)_1_(Dx)_2_, where (D)_1_ and (D)_2_ are the concentrations of UA and PTX that exhibit a determined effect when applied simultaneously to the cells, and (Dx)_1_ and (Dx)_2_ are the concentrations of UA and PTX that exhibit the same determined effect when used in isolation. From this analysis, the combined effects of UA and PTX can be summarized as follows: CI < 1, CI = 1, and CI >1 indicates synergistic, additive, and antagonistic effects, respectively. The isobologram is formed by plotting the individual agent concentrations required to achieve 50% inhibitory effect on their respective x-axes and y-axes. A straight line connecting the two points is drawn, and the concentrations of the two agents used in combination to achieve 50% inhibitory effect are plotted on the isobologram. Combination data points that fall on the line represent an additive interaction, whereas data points that fall below or above the line represent synergism or antagonism, respectively.

### Acridine orange/ethidium bromide dual staining

AO/EB dual staining was used to evaluate the apoptosis-inducing effect. Equal numbers of BGC-823 and SGC-7901 cells (1 × 10^5^) were plated in 24-well dishes, and then incubated with 12 μM UA, 2.5 nM PTX, or a combination of these two agents for 48 hours. The cells were washed in phosphate buffer saline (PBS), and then subjected to trypsin digestion. Cell suspension was transferred to glass clips with the addition of dual fluorescent staining solution (1 μL) containing 100 μg/mL AO and 100 μg/mL EB (Sigma, St. Louis, MO) and subsequently examined by fluorescence microscopy (Eclipse E-800; Nikon, Tokyo, Japan).

### Flow cytometry analysis

For further quantitative analysis of apoptosis, the cells were washed with PBS and stained with annexinV-FITC and propidium iodide (PI) by use of an AnnexinV-FITC kit (Bender Medsystem, Vienna, Austria). The cells were then subjected to flow cytometry according to the manufacturer's instructions, and the stained cells were analyzed by FACScan flow cytometer (Becton Dickinson, CA, USA).

### Western blot analysis

Preparation of cell lysates and sodium dodecyl sulfate-polyacrylamide gel electrophoresis (SDS-PAGE) analysis was performed as described in our previous reports [14, 17]. Primary antibodies used include rabbit anti-COX-2, anti-Bcl-2, anti-Bax (Cell Signaling Technology, Inc., Beverly, MA, USA), anti-PCNA (Santa Cruz Biotechnology, Santa Cruz, CA, USA), and anti-GAPDH (Sigma-Aldrich). Goat anti-rabbit horseradish peroxidase (HRP)-conjugated secondary antibody was purchased from Vector Laboratories Inc. (Burlingame, CA, USA). Bands were quantified by application of densitometric image analysis software (Quantity One, Bio-Rad, Hercules, CA, USA). The relative expressions of COX-2, PCNA, Bcl-2, and Bax were normalized to that of GAPDH.

### Statistical analysis

Statistical analyses were done by SPSS Version 17.0 software (SPSS Inc., Chicago, IL, USA). The data are shown as means ± standard deviation (SD). Statistical comparison among the groups was analyzed by use of ANOVA, followed by Fisher's least significant difference test. *P* < 0.05 was considered statistically significant.

## References

[1] Jemal A, Bray F, Center MM, Ferlay J, Ward E, Forman D (2011). Global cancer statistics. CA Cancer J Clin.

[2] Oh SC (2012). Update of adjuvant chemotherapy for resected gastric cancer. J Gastric Cancer.

[3] Chen W, Zheng R, Baade PD, Zhang S, Zeng H, Bray F, Jemal A, Yu XQ, He J (2016). Cancer statistics in China, 2015. CA Cancer J Clin.

[4] Ishihara R (2010). Infrared endoscopy in the diagnosis and treatment of early gastric cancer. Endoscopy.

[5] Tanizawa Y, Terashima M (2010). Lymph node dissection in the resection of gastric cancer: review of existing evidence. Gastric Cancer.

[6] El Gendy MA, Somayaji V, El-Kadi AO (2010). Peganum harmala L. is a candidate herbal plant for preventing dioxin mediated effects. Planta Med.

[7] Ramsewak RS, DeWitt DL, Nair MG (2000). Cytotoxicity, antioxidant and anti-inflammatory activities of curcumins I-III from Curcuma longa. Phytomedicine.

[8] Limami Y, Pinon A, Leger DY, Mousseau Y, Cook-Moreau J, Beneytout JL, Delage C, Liagre B, Simon A (2011). HT-29 colorectal cancer cells undergoing apoptosis overexpress COX-2 to delay ursolic acid-induced cell death. Biochimie.

[9] Wang W, Zhao C, Jou D, Lü J, Zhang C, Lin L, Lin J (2013). Ursolic acid inhibits the growth of colon cancer-initiating cells by targeting STAT3. Anticancer Res.

[10] Zhang H, Li X, Ding J, Xu H, Dai X, Hou Z, Zhang K, Sun K, Sun W (2013). Delivery of ursolic acid (UA) in polymeric nanoparticles effectively promotes the apoptosis of gastric cancer cells through enhanced inhibition of cyclooxygenase 2 (COX-2). Int J Pharm.

[11] Zhang H, Zheng D, Ding J, Xu H, Li X, Sun W (2015). Efficient delivery of ursolic acid by poly(N-vinylpyrrolidone)-block-poly (epsilon-caprolactone) nanoparticles for inhibiting the growth of hepatocellular carcinoma in vitro and in vivo. Int J Nanomedicine.

[12] Lin J, Chen Y, Wei L, Hong Z, Sferra TJ, Peng J (2013). Ursolic acid inhibits colorectal cancer angiogenesis through suppression of multiple signaling pathways. Int J Oncol.

[13] Yeh CT, Wu CH, Yen GC (2010). Ursolic acid, a naturally occurring triterpenoid, suppresses migration and invasion of human breast cancer cells by modulating c-Jun N-terminal kinase, Akt and mammalian target of rapamycin signaling. Mol Nutr Food Res.

[14] He XP, Shao Y, Li XL, Xu W, Chen GS, Sun HH, Xu HC, Xu X, Tang D, Zheng XF, Xue YP, Huang GC, Sun WH (2012). Downregulation of miR-101 in gastric cancer correlates with cyclooxygenase-2 overexpression and tumor growth. FEBS J.

[15] Sun WH, Sun YL, Fang RN, Shao Y, Xu HC, Xue QP, Ding GX, Cheng YL (2005). Expression of cyclooxygenase-2 and matrix metalloproteinase-9 in gastric carcinoma and its correlation with angiogenesis. Jpn J Clin Oncol.

[16] Chan MW, Wong CY, Cheng AS, Chan VY, Chan KK, To KF, Chan FK, Sung JJ, Leung WK (2007). Targeted inhibition of COX-2 expression by RNA interference suppresses tumor growth and potentiates chemosensitivity to cisplatin in human gastric cancer cells. Oncol Rep.

[17] Sun WH, Zhu F, Chen GS, Su H, Luo C, Zhao QS, Zhang Y, Shao Y, Sun J, Zhou SM, Ding GX, Cheng YL (2008). Blockade of cholecystokinin-2 receptor and cyclooxygenase-2 synergistically induces cell apoptosis, and inhibits the proliferation of human gastric cancer cells in vitro. Cancer Lett.

[18] Prasad S, Yadav VR, Sung B, Gupta SC, Tyagi AK, Aggarwal BB (2016). Ursolic acid inhibits the growth of human pancreatic cancer and enhances the antitumor potential of gemcitabine in an orthotopic mouse model through suppression of the inflammatory microenvironment. Oncotarget.

[19] Li Y, Xing D, Chen Q, Chen WR (2010). Enhancement of chemotherapeutic agent-induced apoptosis by inhibition of NF-kappaB using ursolic acid. Int J Cancer.

[20] Prasad S, Yadav VR, Sung B, Reuter S, Kannappan R, Deorukhkar A, Diagaradjane P, Wei C, Baladandayuthapani V, Krishnan S, Guha S, Aggarwal BB (2012). Ursolic acid inhibits growth and metastasis of human colorectal cancer in an orthotopic nude mouse model by targeting multiple cell signaling pathways: chemosensitization with capecitabine. Clin Cancer Res.

[21] Kavallaris M (2010). Microtubules and resistance to tubulin-binding agents. Nat Rev Cancer.

[22] Huang H, Han Y, Gao J, Feng J, Zhu L, Qu L, Shen L, Shou C (2013). High level of serum AMBP is associated with poor response to paclitaxel-capecitabine chemotherapy in advanced gastric cancer patients. Med Oncol.

[23] Kobayashi M, Oba K, Sakamoto J, Kondo K, Nagata N, Okabayashi T, Namikawa T, Hanazaki K (2007). Pharmacokinetic study of weekly administration dose of paclitaxel in patients with advanced or recurrent gastric cancer in Japan. Gastric Cancer.

[24] Hanna YM, Baglan KL, Stromberg JS, Vicini FA, Decker D (2002). Acute and subacute toxicity associated with concurrent adjuvant radiation therapy and paclitaxel in primary breast cancer therapy. Breast J.

[25] Altorki NK, Keresztes RS, Port JL, Libby DM, Korst RJ, Flieder DB, Ferrara CA, Yankelevitz DF, Subbaramaiah K, Pasmantier MW, Dannenberg AJ (2003). Celecoxib, a selective cyclo-oxygenase-2 inhibitor, enhances the response to preoperative paclitaxel and carboplatin in early-stage non-small-cell lung cancer. J Clin Oncol.

[26] Ferrandina G, Ranelletti FO, Martinelli E, Paglia A, Zannoni GF, Scambia G (2006). Cyclo-oxygenase-2 (Cox-2) expression and resistance to platinum versus platinum/paclitaxel containing chemotherapy in advanced ovarian cancer. BMC Cancer.

[27] Kummar S, Chen HX, Wright J, Holbeck S, Millin MD, Tomaszewski J, Zweibel J, Collins J, Doroshow JH (2010). Utilizing targeted cancer therapeutic agents in combination: novel approaches and urgent requirements. Nat Rev Drug Discov.

[28] Nakanishi K, Kobayashi D, Mochizuki Y, Ishigure K, Ito S, Kojima H, Ishiyama A, Fujitake S, Shikano T, Morita S, Kodera Y (2016). Phase II multi-institutional prospective randomized trial comparing S-1 plus paclitaxel with paclitaxel alone as second-line chemotherapy in S-1 pretreated gastric cancer (CCOG0701). Int J Clin Oncol.

[29] Tuan TF, Tsai ML, Yeh KC, Huang HC, Chung CT, Huang CL, Han CH, Chen CP, Wang MH, Shen CC, Lai YK, Lee WS, Hwang LL, Chen CT (2010). Intravenous paclitaxel against metastasis of human gastric tumors of diffuse type. Cancer Chemother Pharmacol.

[30] Chen Q, Qin R, Fang Y, Li H (2015). Berberine sensitizes human ovarian cancer cells to cisplatin through miR-93/PTEN/Akt signaling pathway. Cell Physiol Biochem.

[31] Zhu RJ, Shen XL, Dai LL, Ai XY, Tian RH, Tang R, Hu YJ (2014). Total aglycones from Marsdenia tenacissima increases antitumor efficacy of paclitaxel in nude mice. Molecules.

[32] Wang X, Zhang F, Yang L, Mei Y, Long H, Zhang X, Zhang J, Qimuge-Suyila Su X (2011). Ursolic acid inhibits proliferation and induces apoptosis of cancer cells in vitro and in vivo. J Biomed Biotechnol.

[33] He K, Song S, Zou Z, Feng M, Wang D, Wang Y, Li X, Ye X (2016). The hypoglycemic and synergistic effect of loganin, morroniside, and ursolic acid isolated from the fruits of cornus officinalis. Phytother Res.

[34] Xiang F, Pan C, Kong Q, Wu R, Jiang J, Zhan Y, Xu J, Gu X, Kang X (2014). Ursolic acid inhibits the proliferation of gastric cancer cells by targeting miR-133a. Oncol Res.

[35] Sun WH, Chen GS, Ou XL, Yang Y, Luo C, Zhang Y, Shao Y, Xu HC, Xiao B, Xue YP, Zhou SM, Zhao QS, Ding GX (2009). Inhibition of COX-2 and activation of peroxisome proliferator-activated receptor gamma synergistically inhibits proliferation and induces apoptosis of human pancreatic carcinoma cells. Cancer Lett.

[36] Sawaoka H, Tsuji S, Tsujii M, Gunawan ES, Sasaki Y, Kawano S, Hori M (1999). Cyclooxygenase inhibitors suppress angiogenesis and reduce tumor growth in vivo. Lab. Lab Invest.

[37] Tsujii M, DuBois RN (1995). Alterations in cellular adhesion and apoptosis in epithelial cells overexpressing prostaglandin endoperoxide synthase 2. Cell.

[38] Kim HJ, Yim GW, Nam EJ, Kim YT (2014). Synergistic effect of COX-2 inhibitor on paclitaxel-induced apoptosis in the human ovarian cancer cell line OVCAR-3. Cancer Res Treat.

[39] Sun K, Tang XH, Xie YK (2015). Paclitaxel combined with harmine inhibits the migration and invasion of gastric cancer cells through downregulation of cyclooxygenase-2 expression. Oncol Lett.

[40] Kim J, Soh SY, Shin J, Cho CW, Choi YH, Nam SY (2015). Bioactives in cactus (Opuntia ficus-indica) stems possess potent antioxidant and pro-apoptotic activities through COX-2 involvement. J Sci Food Agric.

[41] Zhang DQ, Guo Q, Zhu JH, Chen WC (2013). Increase of cyclooxygenase-2 inhibition with celecoxib combined with 5-FU enhances tumor cell apoptosis and antitumor efficacy in a subcutaneous implantation tumor model of human colon cancer. World J Surg Oncol.

[42] Chou TC, Talalay P (1984). Quantitative analysis of dose-effect relationships: the combined effects of multiple drugs or enzyme inhibitors. Adv Enzyme Regul.

[43] Tallarida RJ (2001). Drug synergism: its detection and applications. J Pharmacol Exp Ther.

